# Emodin Alleviates Acute Pancreatitis‐Associated Acute Lung Injury by Inhibiting Serum Exosomal miRNA‐21‐3p‐Induced M1 Alveolar Macrophage Polarisation

**DOI:** 10.1111/jcmm.70758

**Published:** 2025-08-22

**Authors:** Bowen Lan, Xuanchi Dong, Qi Yang, Haiyun Wen, Yibo Zhang, Fan Li, Yinan Cao, Zhe Chen, Hailong Chen

**Affiliations:** ^1^ Department of General Surgery The First Affiliated Hospital of Dalian Medical University Dalian China; ^2^ Laboratory of Integrative Medicine The First Affiliated Hospital of Dalian Medical University Dalian China; ^3^ Department of Urology The Second Affiliated Hospital of Dalian Medical University Dalian China; ^4^ Department of Traditional Chinese Medicine The Second Affiliated Hospital of Dalian Medical University Dalian China; ^5^ Institute (College) of Integrative Medicine Dalian Medical University Dalian China; ^6^ Department of Gastroenterology The 967th Hospital of the Joint Logistics Support Force of the People's Liberation Army of China Dalian China

**Keywords:** acute lung injury, alveolar macrophages, exosome, M1 polarisation, miRNA, severe acute pancreatitis

## Abstract

Severe acute pancreatitis (SAP) is a common abdominal emergency in clinical practice. Approximately 20%–40% of patients with SAP will be associated with acute lung injury (SAP‐ALI), which is a major cause of death. Emodin (EMO) is a naturally occurring anthraquinone derivative with various pharmacological properties. EMO has a therapeutic effect on SAP‐ALI; however, the underlying mechanism remains unclear. Exosomes mediate intercellular communication in disease progression. Therefore, this study aimed to explore the role of serum exosomes in SAP‐ALI and the potential mechanisms by which EMO regulates the composition of exosomes for treatment. miR‐21‐3p was highly expressed in serum exosomes of the SAP group and exacerbated M1 polarisation of alveolar macrophages (AMs). EMO treatment reduced serum exosomal miR‐21‐3p and relatively attenuated M1 polarisation. Transfection with an miR‐21‐3p inhibitor attenuated LPS‐induced M1 polarisation of AMs in vitro; however, its effects were partially reversed when the downstream target gene PTEN was knocked down simultaneously. This study suggests that EMO reduced the enrichment of miR‐21‐3p in serum‐derived exosomes of rats with SAP, inhibiting the M1 polarisation of AMs caused by the transfer of miR‐21‐3p through the exosome pathway. This mechanism is related to miR‐21‐3p targeting of the PTEN/PI3K/AKT signalling pathway.

## 1 Introduction

Acute pancreatitis (AP) is a destructive inflammatory disease of the pancreas caused by the abnormal activation of pancreatic enzymes for various reasons, resulting in the autodigestion of pancreatic tissues. The annual incidence of AP worldwide is 34 cases/100,000 [1]. Most AP cases present as a self‐limiting disease; however, approximately 20% of patients with AP progress to a more severe form of severe acute pancreatitis (SAP), which has a mortality rate of approximately 10‐40% [2, 3]. Systemic inflammatory response syndrome and multiple organ dysfunction syndrome (MODS) are the main features of lethality in SAP [4, 5]. Acute lung injury (ALI) is a major complication in patients with SAP, with an incidence of approximately 30% [6]. When ALI was accompanied by SAP, the mortality rate increased to 50% [7]. Despite increasing research on SAP‐ALI in recent years, its pathogenesis has not been fully clarified. Therefore, it is essential to explore the pathogenesis of SAP‐ALI and identify new targets for gene therapy to develop clinical strategies.

Macrophages are resident immune cells in the lungs and are known as alveolar macrophages (AMs) when they travel through the alveolar lumen. AMs are the primary interest‐free cell population in the alveolar lumen. They maintain a high alertness to foreign bodies and pathogens under healthy physiological conditions. They can clear dust particles, bacteria, cell debris and even tumour cells from the alveoli and pulmonary interstitium, thus playing an essential role in the immune defence. However, AMs are overactivated in the disease state [8]; the uncontrolled inflammatory response forms a storm of inflammatory factors, which in turn damages vascular endothelial cells and alveolar epithelial cells, leading to ventilation/exchange dysfunction of the lung, and is an important factor in the formation of ALI. An imbalance in AM polarisation is one of its important forms of activation [9]. The commonly studied phenotypes of AM polarisation include classically activated macrophages (M1) and alternatively activated macrophages (M2). M1 macrophages release pro‐inflammatory factors and promote inflammatory responses and phagocytosis, whereas M2 macrophages release anti‐inflammatory factors, inhibit inflammatory responses and promote tissue repair. When M1 macrophages excessively accumulate in the lung, their secretion of pro‐inflammatory mediators such as tumour necrosis factor‐alpha (TNF‐α), interleukin (IL)‐6 (IL‐6), IL‐1 and inducible nitric oxide synthase (iNOS) amplifies the immune response, causing inflammatory storms leading to ALI [10].

Exosomes are extracellular vesicles with a diameter of approximately 30–150 nm and are composed of proteins, nucleic acids, lipids and other components. As a new type of intercellular communication, exosomes can carry specific contents (proteins, microRNAs [miRNAs], long noncoding RNAs [lncRNAs]) to reach and deliver them to target cells, thereby regulating the physiological progression of the disease [11]. miRNAs are a class of short‐stranded noncoding RNAs that are approximately 24 nucleotides long and are widely enriched in exosomes [12]. Under pathological conditions, most tissues and cells selectively secrete specific miRNAs, which can be detected in circulating exosomes [13, 14, 15]. When circulating exosomes loaded with these specific miRNAs are delivered to target cells, they usually alter the target cell phenotype by negatively regulating downstream target genes [16], thus participating in disease regulation. Sequencing data from previous studies demonstrated that miR‐21‐3p was overexpressed in the pancreatic acinar cells of C57BL/6 mice with AP [17] and miR‐21‐3p exacerbated acute haemorrhagic necrotising pancreatitis in Sprague–Dawley (SD) rats [18]. In addition, serum expression of miR‐21‐3p has been shown to be positively correlated with the severity of AP and ALI in clinical trials [19, 20]. Thus, miR‐21‐3p appears to be an important mediator of SAP‐ALI.

Currently, the treatment of SAP‐ALI mainly involves treating the primary disease, providing respiratory support and administering anti‐inflammatory drug therapy. However, the clinical treatment of SAP‐ALI has yet to achieve the desired therapeutic effect owing to the lack of specific modalities, and mortality rates remain high. Therefore, researchers have sought treatments with fewer adverse effects and with improved efficacy. Traditional Chinese medicine (TCM) has been used for thousands of years and is characterised by fewer side effects and multitarget modulation. Among them, emodin (EMO), a naturally occurring anthraquinone derivative, is an active substance in Chinese herbal medicines, such as rhubarb, He Shou Wu, *Aloe vera* and cassia seed, and possesses a wide range of pharmacological effects, including anticancer, anti‐inflammatory, antioxidant and antibacterial activities [21]. Many previous studies have confirmed the therapeutic effects of EMO in SAP‐ALI through various pathways, including inhibition of AMs pyroptosis [22], inhibition of NLRP3 inflammatory vesicles and NF‐κB activation [23, 24], inhibition of neutrophil protease activity [25] and strengthening the alveolar epithelial cell barrier [26]. Recent findings confirmed that changes in miRNAs in circulating exosomes are a way for EMO to exert its therapeutic effects [27].

The current study aimed to elucidate whether EMO can treat SAP‐ALI via the serum‐derived exosomal miRNA pathway. We confirmed the high expression of exosomal miR‐21‐3p in the serum of SAP rats. During SAP, exosomal miR‐21‐3p promotes ALI by inducing M1 polarisation of AMs. EMO treatment effectively alleviated ALI by reducing serum‐derived exosomal miR‐21‐3p expression. In addition, we explored the mechanism by which miR‐21‐3p promotes the onset of M1 polarisation in AMs to provide new avenues for the treatment of SAP‐ALI.

## 2 Materials and Methods

### 2.1 Animals

Healthy male SD rats were obtained from Liaoning Changsheng Biological Co. LTD, aged 6–8 weeks, weighing 180–220 g. All experimental animals complied with the regulations of the People's Republic of China on the use and management of experimental animals during the experiments and were approved by the Experimental and Animal Ethics Committee of Dalian Medical University (Approval number: AEE19003). The experimental animals were housed in individually ventilated cages (Suhang Technology Equipment, Suzhou, China) at an ambient temperature of 22°C ± 2°C, a humidity of 50%± 10%, a 12‐h light–dark cycle, and were allowed to eat and drink ad libitum.

### 2.2 Establishment of SAP Rat Model and Experimental Design

Twenty‐four SD rats were randomly divided into three groups (*n* = 8 each): sham operation (SO), SAP and EMO. They fasted for 12 h before surgery but had free access to water. On the day of surgery, the rats were weighed, and isoflurane (R510‐22‐10; RWD, Shenzhen, China) was administered for inhalational induction of anaesthesia using a small animal respiratory anaesthesia machine (R500; RWD). After skin preparation in the subxiphoid surgical area, the rats were fixed in the supine position on the operating table and a mask was attached for low‐flow continuous anaesthesia. In the SO group, the rats developed pancreatitis several times and the abdomen was closed. In the SAP group, the SAP‐ALI rat model was established by the retrograde injection of 5% sodium taurocholate (STC) into the pancreaticobiliary duct (50 mg/kg; Cat.#T8510; Solarbio, Beijing, China) [25]. STC was slowly pumped into the pancreaticobiliary duct with a micropump at the rate of 10 μL/min and left for 3 min after the completion of the injection. The EMO group was administered EMO (40 mg/kg) via gastric gavage at 2 and 12 h after SAP modelling [25]. After 24 h, all animals were sacrificed after blood sampling from the abdominal aorta under anaesthesia. The whole blood, bronchoalveolar lavage fluid (BALF), lung tissues and pancreas tissues were collected from each rat in the order of modelling for subsequent experiments.

To study the effect of exosomes on rats with SAP‐ALI, an additional 40 SD rats were randomly divided into five groups (*n* = 8 each): SO, SAP, CON‐Exo, SAP‐Exo and EMO‐Exo. The SO and SAP groups were treated as described above, and the CON‐Exo, SAP‐Exo and EMO‐Exo groups were injected with exosomes (250 μg/rat) [28, 29] isolated from the serum of the corresponding subgroups by tail vein injection 2 h after modelling. After 24 h, whole blood, BALF, lung tissue and pancreatic tissue samples were collected from each rat.

### 2.3 Cell Culture and Experimental Design

The rat alveolar epithelial cell line NR8383 was purchased from Procell Life Science & Technology Co. Ltd. (Wuhan, China). The cells were cultured in Ham's F12K (Macgene, Cat.#10025) medium containing 20% fetal bovine serum (FBS, Gibico, NY, USA) and 1% triple antibiotics (KeyGEN, Cat.#KGY0073, Nanjing, China) in a cell culture incubator at 37°C and 5% CO_2_. To demonstrate the effect of exosomes on the M1 polarisation of AMs, NR8383 cells were co‐cultured with equal amounts of exosomes from each treatment group. The experiments were divided into five groups: control (CON), lipopolysaccharide (LPS), LPS + SO‐Exo (SO‐Exo), LPS + SAP‐Exo (SAP‐Exo) and LPS + EMO‐Exo (EMO‐Exo). Well‐conditioned NR8383 cells were cultured in 10 cm dishes overnight 1 day before the experiment to allow the cells to grow uniformly against the wall. The next day, the M1 polarisation of NR8383 cells was induced by stimulation with 1 μg/mL LPS (Sigma‐Aldrich, Cat.#L4516) in the blank medium for 24 h [30]. Next, exosomes (50 μg/mL) [31] from each treatment group were added to the culture medium after 2 h of LPS stimulation and co‐cultured until 24 h. The supernatants and cells were collected separately for subsequent experiments.

In addition, to demonstrate the mechanism by which miR‐21‐3p promotes the M1 polarisation of AMs, cells were transfected with an miR‐21‐3p inhibitor and siPTEN. Specific experiments were performed to divide NR8338 macrophages into six groups: CON, LPS, LPS + miR‐21‐3p inhibitor negative control (NC), LPS + miR‐21‐3p inhibitor, LPS + miR‐21‐3p inhibitor + siNC and LPS + miR‐21‐3p inhibitor + siPTEN.

### 2.4 Cell Transfection

The miR‐21‐3p mimics/inhibitor and NC, and siRNA were purchased from GenePharma (Suzhou, China). Cells were strictly transfected according to the manufacturer's instructions. Briefly, NR8338 cells were transfected using the GP‐transfect‐Mate transfection reagent (GenePharma, Cat.#G04008) in Ham's F12K medium without serum or the triple antibody. According to the manufacturer's instructions, NR8383 cells were transfected with the mimics, inhibitor, or their respective NC at a final concentration of 50 pmol/mL. After 6 h of transfection, the complete medium was replaced, and the cells were incubated for up to 24 h. The three PTEN siRNAs were designed by GenePharma. Twenty‐four hours after siPTEN transfection, quantitative real‐time PCR (RT‐qPCR) was used to detect the knockdown efficiency of PTEN, and the sequence with the highest knockdown efficiency was selected for subsequent experiments.

### 2.5 Isolation and Characterisation of Exosomes

After the arterial blood was removed, it was allowed to stand for 1 h and then centrifuged (4°C, 3000 rpm) for 15 min to collect the serum. The serum was first centrifuged for 50 min (4°C, 5000 g) and then filtered through a 0.45‐μm sterile filter membrane for exosome isolation. Exosome isolation and purification were performed using a kit based on the principle of size‐exclusion chromatography (RENGEN BIOSCIENCE, Cat.#EXOSECon1.0–3), according to the manufacturer's instructions. The obtained exosomes were stored at −80°C.

Transmission electron microscopy (TEM): the exosome suspension was diluted to a suitable magnification, and 20 μL of sample was pipetted onto a copper mesh and precipitated for 3–5 min. Subsequently, 2% phosphotungstic acid was dropped onto the copper mesh and precipitated for 2 min. The excess liquid was removed with filter paper, and the copper mesh was allowed to dry at room temperature before being placed under a transmission electron microscope (JEM1400PLUS, Japan) for image acquisition.

Nanoparticle tracking analysis (NTA): Exosomes were diluted 1000‐fold in enzyme‐free water and subjected to a particle size assay using an NTA particle size detector (Malvern, UK). Each sample was averaged over three measurements for statistical analysis.

Western blotting (WB): the expression of exosome‐positive protein markers (CD81, CD63 and TSG101) and the negative protein marker calnexin was analysed.

### 2.6 Exosome Tracing In Vivo and In Vitro

Fluorescent labeling was performed using 100 μM of red exosome fluorescent dye PKH‐26 working solution (Umbio, Cat.#UR52302, Shanghai, China) incubated with exosomes under light‐avoidance conditions for 10 min. Excess fluorescent dye was removed using ultrafiltration tubes. Finally, the fluorescently labelled exosomes were resuspended in sterile PBS. In vivo, PKH‐26 fluorescently labelled exosomes (250 μg/rat) were injected into rats via the tail vein. After 24 h, frozen sections of lung tissues were prepared, and the nuclei of the cells were re‐stained with DAPI (Solarbio, Cat.#C0065) before being placed under a fluorescence microscope (Olympus, Japan) and photographed. In vitro, when NR8383 was cultured to a suitable density, PKH‐26 fluorescently labelled exosomes were co‐cultured with NR8383 cells (50 μg/mL) under light protection for 24 h, then after fixation by 4% precooled paraformaldehyde fixative (PFA, JIJIA Biotechnology, Cat.#J2001) and DAPI re‐staining of the cell nuclei, the exosomes were examined for their uptake by the cells under a fluorescence microscope.

### 2.7 Histopathological Analysis

The animal tissues were removed and placed in 4% neutral PFA, left overnight to fix, then embedded in paraffin and cut into 4‐μm sections. The sections were dewaxed, rehydrated and stained with haematoxylin and eosin (H&E). Finally, tissue sections were dehydrated and mounted on slides.

### 2.8 Pathological Score

The H&E‐stained sections were observed under a light microscope, and three fields were randomly selected for pathological scoring. Pancreatic histopathological scoring was based on the approach of Kusshe et al. [32] Four aspects of pancreatic tissue edema, inflammatory cell infiltration, haemorrhage and necrosis were assessed with a score of 0–4 for each, and then the total of the four scores was taken as the pathological score for one field, and the average of the pathology scores of the three fields of view was taken as the final pathology score of one sample. The criteria for assessing ALI were based on the recommendations of the American Thoracic Society [33, 34]. Histopathological scoring of lungs was performed as described by Leiphrakpam et al. Three aspects of alveolar edema, intra‐alveolar haemorrhage and inflammatory cell infiltration were scored on a scale of 0–3 respectively. The algorithm for scoring each sample was the same as that used for the pancreas [35].

### 2.9 Serum Amylase

Serum amylase (AMY) was assayed using an amylase kit (Jiancheng, Cat.#C016‐1‐1, Nanjing, China) for the starch‐iodine colorimetric assay. Serum was diluted in appropriate proportions and added to excess and known concentrations of the substrate buffer, followed by an iodine solution, which was allowed to bind to the unhydrolysed starch in the sample to form a blue complex. Finally, AMS viability was calculated by measuring the absorbance of the blue complex at 660 nm using an enzyme marker (BioTek instrument, America).

### 2.10 Lung Wet/Dry Weight Ratio

The wet/dry (W/D) ratio was measured in the right upper lung of all rats. The right upper lung was removed and immediately weighed after drying the surface with filter paper. They were then placed in an oven (Thermo, American) at 80°C and weighed after complete drying. Finally, the wet weight was divided by the dry weight to obtain the W/D ratio.

### 2.11 BALF Protein Concentration Assay

After the rats were anaesthetised, the right main bronchus was ligated and the left lung was lavaged three times with 5 mL of precooled saline via tracheal intubation. The obtained BALF was centrifuged (4°C, 1000 g) for 5 min and the supernatant was placed at −80°C for storage. The protein concentration in the BALF was measured using a BCA protein concentration kit (KeyGEN, Cat.#KGP902). After the reaction, the absorbance of the reactant was measured using an enzyme‐labelled instrument at 562 nm, and a standard curve was drawn to calculate the protein concentration of the sample to be examined.

### 2.12 Serum/BALF Inflammatory Factors

Inflammatory factors (IL‐6 and TNF‐α) in the serum and BALF were detected using a double‐antibody sandwich ELISA kit (Westang, Cat.#F15870/F16960, Shanghai, China). The serum and BALF samples were prepared according to the instructions of the kit, and after the reaction was completed, the absorbance was measured at 450 nm using an enzyme‐labelled instrument, and the concentration of the inflammatory factors in the samples to be tested was calculated by plotting the calibration curve.

### 2.13 WB Analysis

Total lung tissue and cellular proteins were extracted using a whole protein extraction kit (KeyGEN, Cat.#KGP2100) according to the manufacturer's instructions from the reagent vendor. Protein concentration was quantified using a BCA protein quantification kit (KeyGEN, Cat.#KGP902), and the total protein concentration was adjusted to the same level. Next, an appropriate amount of 5× loading buffer (Solarbio, Cat.#P1041) was added to the protein samples and boiled for 5–10 min to denature the proteins, followed by centrifugation at 1200 rpm for 5 min, and the supernatant was subjected to electrophoresis.

Depending on the molecular weight of the target proteins, electrophoresis was performed using sodium dodecyl sulfate‐polyacrylamide gel electrophoresis (SDS‐PAGE) gels at a concentration of 8%–10%, after which the proteins were transferred onto 0.22 μm PVDF membranes. After transfer, the membrane was blocked with 5% skim milk at room temperature for 2 h. After washing the membranes with 1 × TBST, they were incubated with primary antibodies overnight at 4°C: Anti‐PTEN (1:1000, Abclonal, Cat.#A11128), Anti‐CD86 (1:1000, HUABIO, Cat.#ET1606‐50), Anti‐iNOS (1:800, Abclonal, Cat.#A0312), Anti‐p‐PI3K (1:500, Bioss, Cat.#bs‐3332R), PI3K (1:800, Zenbio, Cat.#R22768), AKT (1:1000, Proteintech, Cat.#10176–2‐AP), p‐AKT (1:1000, Abmart, Cat.#T40067), β‐actin (1:50,000), CD81 (1:1000, Abcnonal, Cat.#A4863), CD63 (1:1000, Abcnonal, Cat.#A19023), Calnexin (1:1000, Affinity, Cat.#AF5382), TSG101 (1:1000, HUABIO, Cat.#ET1701‐59). The next day, 1 × TBST was used to wash off the excess primary antibody on the membranes, which were then incubated at room temperature with HRP goat anti‐rabbit IgG (H + L) or HRP goat anti‐mouse IgG (H + L) secondary antibody (1:8000, Abways, Cat.# AB0101/AB0102) for 1 h, followed by 1 × TBST to wash away the excess secondary antibody. Protein bands were visualised using an automatic chemiluminescence image analyser (Tanon4800, China), and grey‐level analysis was performed using ImageJ.

### 2.14 RNA Preparation and RT‐qPCR

First, according to the manufacturer's instructions, TRnaZol (NCM, Cat#M5101, Suzhou, China) was used to extract mRNA from tissues and cells, and an miRNA extraction kit (Vazyme, Cat.#RC201, Nanjing, China) was used to extract total miRNA from tissues and cells. Next, 1 μg mRNA or 500 ng miRNA was reverse transcribed into cDNA using MonScript RT III All‐in‐One Mix with dsDNase (Monad, Cat.#MR05101, Suzhou, China) and Evo M‐MLV Reverse Transcription Kit II (Accurate Biology, Cat.#AG11711, Hunan, China). We used the stem‐loop method for reverse transcription of miRNA. The cDNA was then specifically amplified with MonAmp RapidStart Universal SYBR Green qPCR Mix (Monad, Cat.#MQ10701S, Suzhou, China) for specific amplification and quantitative detection using a PCR detection system (BIOER, China). The reaction programme for amplification was as follows: 95°C for 1 min, 40 cycles for 10 s at 95°C, 60°C and 10 s. mRNA expression was analysed using GAPDH as an internal reference, and miRNA expression was analysed using U6 as an internal reference. All data were analysed by the 2^−ΔΔCT^ method for relative quantitative analysis. The primer sequences are listed in Table 1 below.

**TABLE 1:** Primer sequences used for RT‐qPCR.

Gene	Primer	Sequence
miR‐21‐3p	Forward	ATCGCAACAGCAGTCGATGG
	Reverse	AGTGCAGGGTCCGAGGTATT
	Stem‐loop	GTCGTATCCAGTGCAGGGTCCGAGG TATTCGCACTGGATACGACGACAGC
PTEN	Forward	ACCGCCAAATTTAACTGC
	Reverse	GTCTTCACTTAGCCATTGGTC
CD86	Forward	ATAACGTGAATGCCAAGTACCTG
	Reverse	AATCATACAAGCCCGTGTCC
iNOS	Forward	GGATGTGGCTACCACTTTGA
	Reverse	CATGATAACGTTTCTGGCTCTTG
U6	Forward	GGAACGATACAGAGAAGATTAGC
	Reverse	TGGAACGCTTCACGAATTTGCG
miR‐21‐3p mimics	Forward	CAACAGCAGUCGAUGGGCUGUC
	Reverse	CAGCCCAUCGACUGCUGUUGUU
miR‐21‐3p inhibitor		GACAGCCCAUCGACUGCUGUUG
GAPDH	Forward	CTGGAGAAACCTGCCAAGTATG
	Reverse	GGTGGAAGAATGGGGAGTTGCT

### 2.15 Immunofluorescence Staining

For immunofluorescence of NR8383 cells, the cells were cultured in 6‐well plates to a suitable density and fixed with precooled 4% PFA for 10 min. An appropriate amount of DAPI was then added dropwise to cover the bottom of the dish, and the nuclei of the cells were stained in the dark for 5 min. Finally, an anti‐fluorescence quencher was added dropwise and the cells were photographed under a fluorescence microscope.

For immunofluorescence of lung tissue, the sections were co‐incubated with the primary antibodies anti‐CD68 (1:250, Servicebio, Cat. #GB113109) and anti‐iNOS (1:500, Abcnonal, Cat. #A3774) at 4°C overnight. The next day, sections were incubated with the appropriate fluorophore‐conjugated secondary antibodies (1:200, Servicebio, Cat.#AS014). DAPI was used for nuclear counterstaining.

### 2.16 Dual Luciferase Gene Reporter Assay

The Renilla/firefly luciferase reporter plasmids pmirGLO containing wild‐type (WT) or mutant (Mut) PTEN 3′‐UTR along with miR‐21‐3p mimics or miR‐NC were co‐transfected into NR8383 cells. After 48 h of transfection, the cells were collected, and firefly and Renilla luciferase activities were evaluated using the dual‐luciferase reporter assay system (Promega, Madison, USA).

### 2.17 Statistical Analysis

All experimental values are expressed as mean ± standard deviation (SD) and statistically analysed using GraphPad Prism 9.0. An unpaired *t*‐test was used to compare two groups, and one‐way analysis of variance (ANOVA) was used to compare multiple groups. *p* value < 0.05 indicated a statistical difference between two groups.

## 3 Results

### 3.1 Treatment With EMO Attenuated SAP‐ALI

**FIGURE 1:**
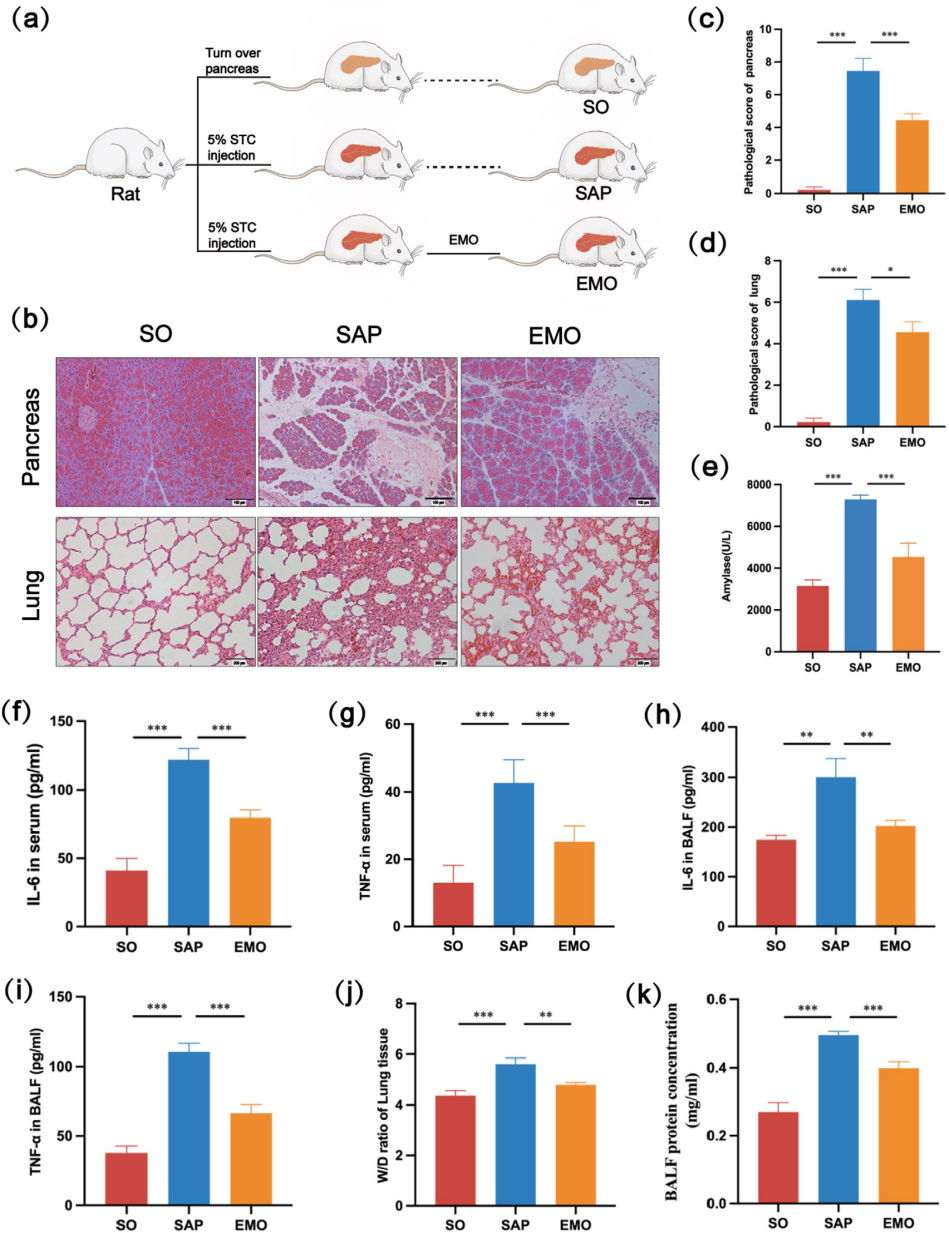
Treatment with EMO effectively alleviated SAP‐ALI in rats. (a) Schematic representation of animal grouping and treatment. (b) Representative histopathological images of pancreas and lungs from each group (scale bar: 100 μm). (c) Pathological score of pancreatic tissues in each group. (d) Pathological score of lung tissues in each group. (e) Levels of serum AMY in each group. (f, g) Levels of IL‐6 and TNF‐α in serum of each group. (h, i) Levels of IL‐6 and TNF‐α in BALF of each group. (j) W/D ratio of lung tissues in each group. (k) Protein concentration in BALF of each group.

To verify the successful construction of the SAP‐ALI rat model and the therapeutic effect of EMO, we first observed pancreatic and lung histopathology and detected serum AMY. Compared to the SO group, the pancreatic pathology in the SAP group showed liquefactive necrosis of the pancreatic tissue, diffuse inflammatory cell infiltration and haemorrhage, and increased pancreatic pathology scores. At 24 h after modelling, rats exhibited typical ALI. Lung histopathology showed thickening and fracture of the alveolar septum, interstitial edema, haemorrhage and inflammatory cell infiltration, and an increased lung injury score. AMY is the first biochemical indicator for the diagnosis of acute pancreatitis. It was significantly elevated in the SAP group and was approximately threefold higher than that in the SO group. However, compared to the SAP group, the pancreatic and lung pathology of the rats treated with EMO by gavage showed that tissue damage was alleviated (Figure 1b), and the pathological scores and serum amylase content were decreased (Figure 1c–e). Next, to assess systemic as well as lung inflammation in rats, we measured the levels of the inflammatory factors TNF‐α and IL‐6 in serum and BALF. The results showed that IL‐6 and TNF‐α levels were significantly elevated in serum and BALF in the SAP group compared to the SO group, and both were reduced after EMO treatment (Figure 1f–i). Finally, we assessed the degree of lung tissue edema and pulmonary microvascular leakage by examining the W/D ratio of the lung tissue and protein concentration in BALF. The results showed that both the lung tissue W/D ratio and protein concentration in BALF were relatively elevated in the SAP group compared to those in the SO group, and both were reduced to some extent after EMO treatment (Figure 1j,k). Taken together, we demonstrated that the SAP‐ALI rat model was successfully established by retrograde injection of 5% STC into the pancreaticobiliary duct and that EMO had a significant therapeutic effect on SAP‐ALI.

### 3.2 EMO Attenuates SAP‐ALI by Reducing M1 Polarisation of AMs

**FIGURE 2:**
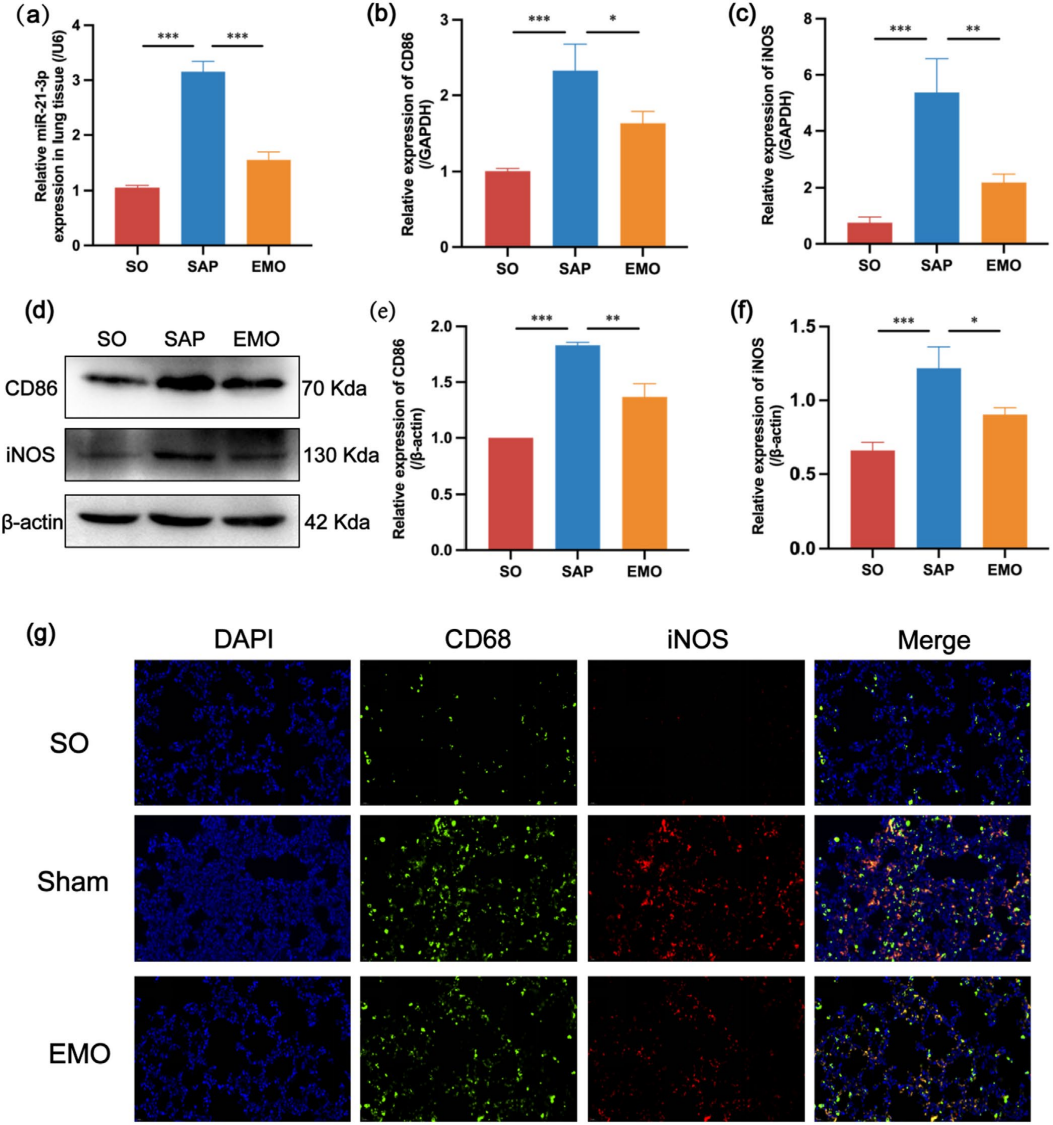
EMO may inhibit M1 polarisation of AMs by reducing miR‐21‐3p expression in lung tissue to treat SAP‐ALI. (a) RT‐qPCR to detect gene expression levels of miR‐21‐3p in lung tissues of each group. (b, c) RT‐qPCR results of gene expression levels of CD86 and iNOS in lung tissues of each group. (d) WB results of CD86 and iNOS in lung tissues of each group. (e, f) Relative quantification of CD86 and iNOS protein expression in WB results. (g) Representative immunofluorescence images of CD68 (green) and iNOS (red) (scale bar: 20 μm).

To verify the effect of EMO treatment on the expression levels of miR‐21‐3p and M1 polarisation of AMs in the lung tissue, we detected the expression of miR‐21‐3p in the lung tissues of each group using RT‐qPCR and the expression of two markers (CD86 and iNOS) in M1 AMs using RT‐qPCR and WB respectively. The results showed that the expression levels of miR‐21‐3p in the lung tissue of SAP rats were higher than that in the SO group and decreased after EMO treatment (Figure 2a). The expression of CD86 and iNOS at the mRNA and protein levels was consistent with that of miR‐21‐3p (Figure 2b–f). In addition, the polarisation level of M1‐type AMs was further confirmed by double fluorescent staining for CD68 (marking AMs) and iNOS (marking M1‐type AMs) (Figure 2g). In general, EMO may inhibit the M1 polarisation of AMs by inhibiting the overexpression of miR‐21‐3p in the lung tissue to exert a therapeutic effect on ALI.

### 3.3 Isolation and Characterisation of Exosomes

**FIGURE 3:**
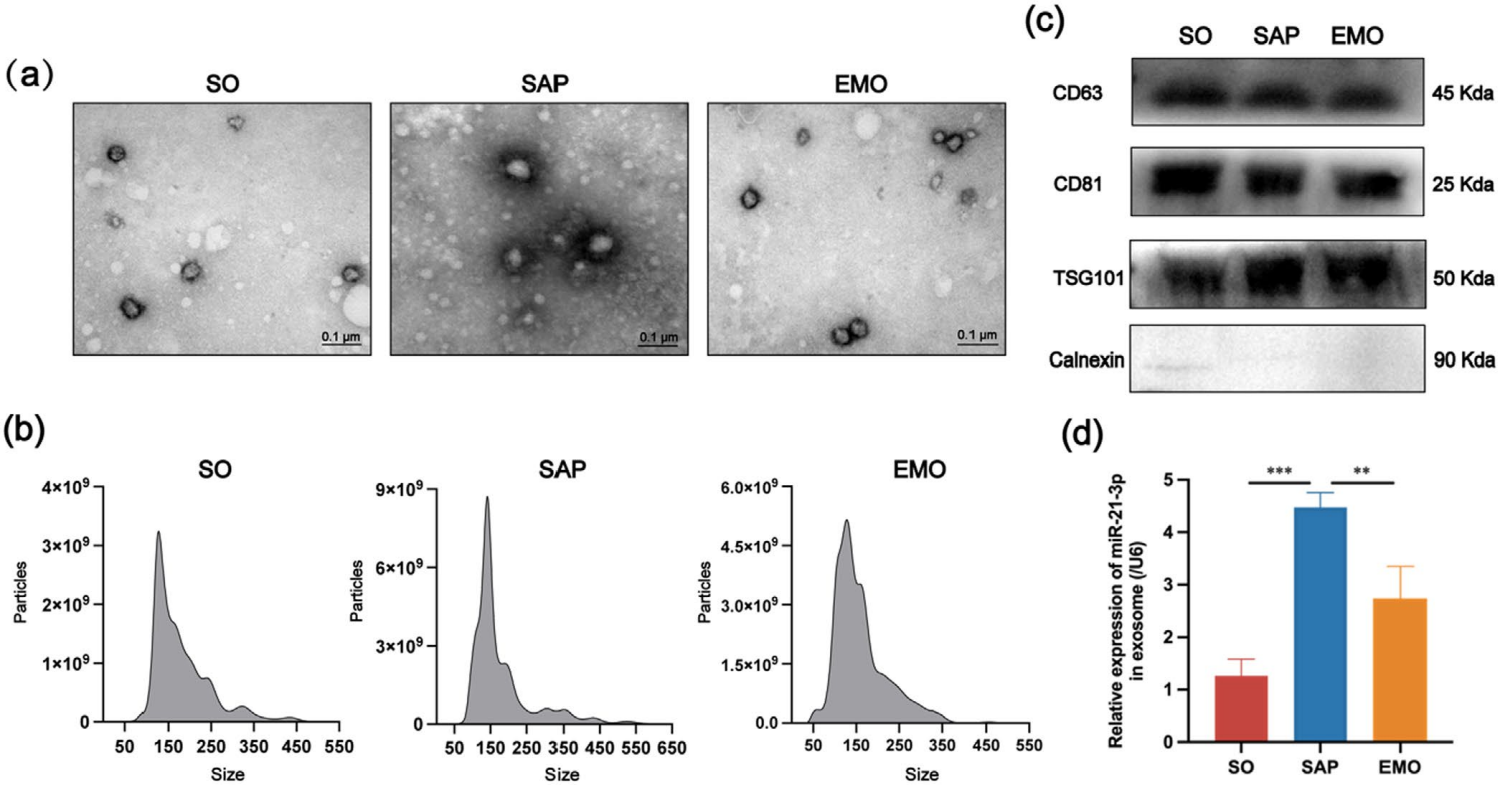
Characterisation of serum‐derived exosomes and miR‐21‐3p expression in each group. (a) Morphological characterisation of serum‐derived exosomes using TEM (scale bar: 0.1 μm). (b) Distribution of serum exosome particle diameter using NTA. (c) WB results of exosome markers CD63, CD81, TSG101 and calnexin (*n* = 3). (d) Expression levels of miR‐21‐3p in serum‐derived exosomes of each group.

To prove that exosomes were successfully isolated, we detected four exosome protein markers by WB, and the morphology and particle size by TEM and NTA respectively. TEM showed the exosomes to be round (Figure 3a). The NTA results show that the particle diameter was approximately 150 nm (Figure 3b). The WB results showed that the exosomes isolated from the serum of each group expressed the three positive markers, CD63, CD81 and TSG101, and hardly expressed the negative marker, calnexin (Figure 3c). The exosome characteristics detected using the above three techniques met the criteria of the Extracellular Vesicle Association for exosomes, which can demonstrate successful exosome extraction.

Previous studies have shown that miR‐21‐3p is closely related to the occurrence of cellular inflammation, especially pulmonary inflammation, mediated by M1 polarisation of AMs [36, 37, 38]. This result was confirmed in our previous study. As a kind of intercellular interaction medium, exosomes transport miRNAs contained within to recipient cells to participate in the occurrence and development of diseases. Therefore, to confirm whether miR‐21‐3p in the exosome pathway was affected during SAP‐ALI, we examined the expression of serum‐derived exosomal miR‐21‐3p in each treatment group using RT‐qPCR. As shown in Figure 3d, the expression levels of serum‐derived exosomal miR‐21‐3p were significantly increased in the SAP group compared to the SO group, whereas they decreased after EMO treatment compared to the SAP group. Thus, we speculate that EMO may play a therapeutic role in SAP‐ALI by reducing the expression levels of miR‐21‐3p in the lung tissue by reducing exosomal miR‐21‐3p, thereby inhibiting the M1 polarisation of AMs.

### 3.4 Serum‐Derived Exosomes Promote M1 Polarisation of AMs via miR‐21‐3p In Vivo

**FIGURE 4:**
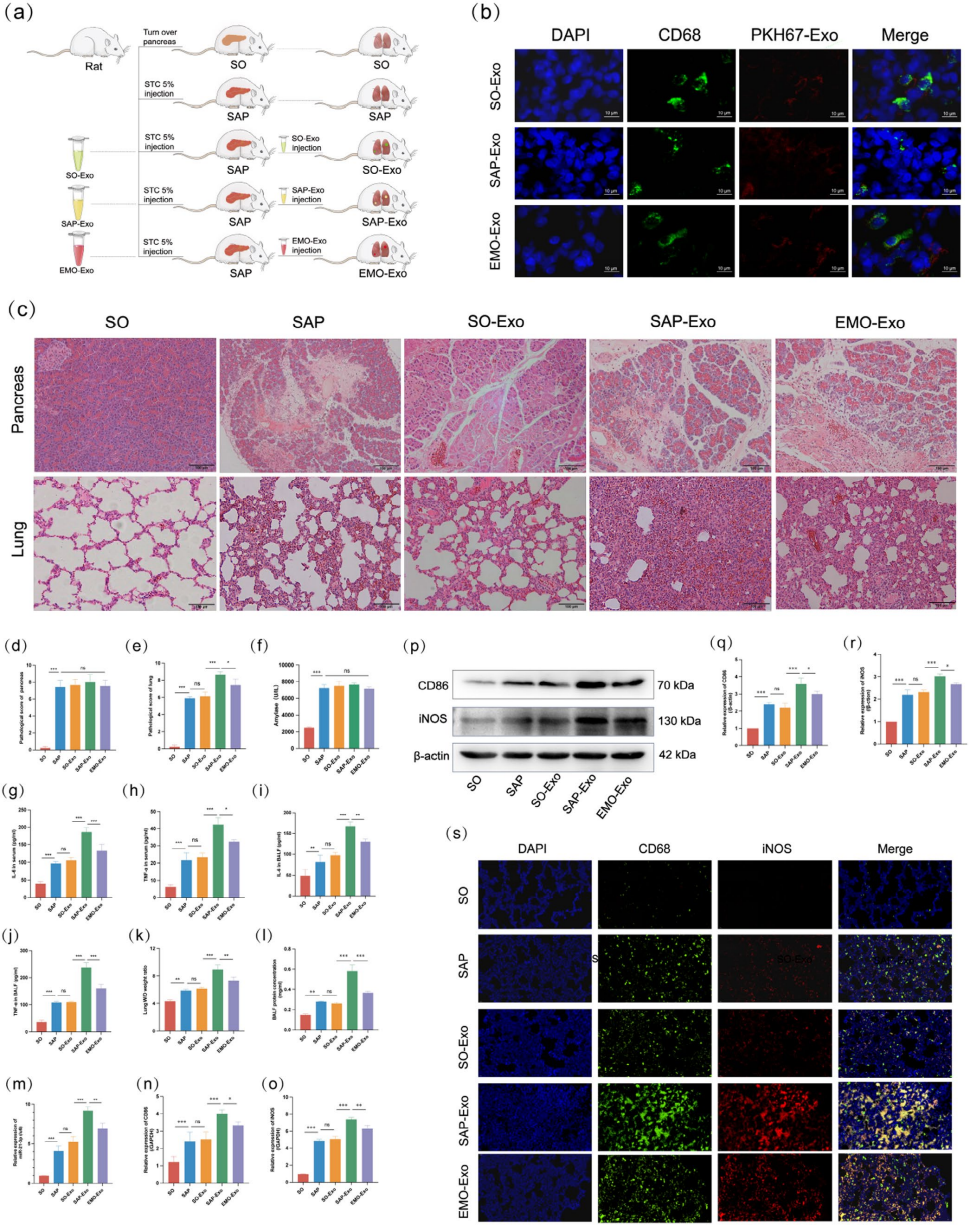
Serum‐derived exosomes promote M1 polarisation of AMs via miR‐21‐3p in vivo. (a) Schematic representation of animal grouping and treatment. (b) Tracing of PKH‐26 fluorescently labelled exosomes in the AMs (scale bar: 10 μm). (c) Representative pancreatic and lung histopathology of each group (scale bar: 100 μm). (d) Pathological score of pancreatic tissues in each group. (e) Pathological score of lung tissues in each group. (f) Serum AMY levels in each group. (g, h) Levels of IL‐6 and TNF‐a in serum of each group. (i, j) Levels of IL‐6 and TNF‐α in BALF of each group. (k) W/D ratio of lung tissues in each group. (l) Protein concentration in BALF of each group. (m) eEpression levels of miR‐21‐3p in lung tissues of each group. (n) WB results of CD86 and iNOS in lung tissue of each group. (o, p) Relative quantification of CD86 and iNOS gene expression in lung tissues of each group (*n* = 3). (q, r) Relative quantification of CD86 and iNOS protein expression in WB results. (s) Representative immunofluorescence images of CD68 (green) and iNOS (red) (scale bar: 20 μm).

As shown in Figure 4a, to investigate whether serum‐derived exosomal miR‐21‐3p causes ALI by inducing M1 polarisation of AMs at the onset of SAP, we injected exosomes isolated from the serum of animals in the SO, SAP and EMO groups into rats with SAP via tail vein injection, which were named as SO‐Exo, SAP‐Exo and EMO‐Exo groups respectively. The SO and SAP groups were used as the SO groups.

First, to confirm that the exosomes reached the AMs, we injected PKH67 fluorescently labelled exosomes into the rat circulatory system via the tail vein, and they were captured in the AMs by immunofluorescence 24 h after injection (Figure 4b). Next, the extent of pancreatic and lung tissue damage was examined. H&E staining showed significant oedema, necrosis, haemorrhage and inflammatory cell infiltration in the pancreatic tissues of the remaining four groups compared to the SO group, but there was no significant difference in the pathological scores (Figure 4c,d). Similarly, compared to the SO group, histopathological changes in the lung tissues of the four model groups were obvious, showing a thickened alveolar septum, significantly reduced alveolar space, and scattered red blood cells and neutrophils. There was no significant difference in pathological damage between the SO‐Exo and SAP groups. Compared with the SO‐Exo group, the pathological damage in the SAP‐Exo and EMO‐Exo groups was aggravated, but tissue damage in the EMO‐Exo group was alleviated compared with that in the SAP‐Exo group (Figure 4e). Serum AMY levels were consistent with the degree of pathological pancreatic damage (Figure 4f). The levels of inflammatory factors (IL‐6 and TNF‐α) in the serum and BALF confirmed that SO‐Exo did not have a significant effect on systemic and lung inflammation in SAP rats. The SAP‐Exo group showed a significant increase in systemic and lung inflammation, whereas the degree of elevated inflammation in the EMO‐Exo group was lower than that in the SAP‐Exo group (Figure 4g–j). The lung tissue W/D ratio and protein concentration in the BALF were consistent with the degree of pulmonary inflammation (Figure 4k,l).

To further confirm the effect of exosomal miR‐21‐3p on the level of M1 polarisation in AMs, we examined the expression level of miR‐21‐3p in lung tissues using RT‐qPCR, and the expression levels of CD86 and iNOS, two M1 macrophage markers, by WB and RT‐qPCR. The results showed that the level of miR‐21‐3p in the lung tissue of the SO‐Exo group was not significantly different from that in the SAP group. The expression of miR‐21‐3p in the lung tissue of the SAP‐Exo group was significantly higher than that in the SO‐Exo group, whereas the expression in the EMO‐Exo group was lower than that in the SAP‐Exo group (Figure 4m). The expression levels of iNOS and CD86 mRNA and protein in the lung tissues of each group were consistent with the changes in miR‐21‐3p levels in the lung tissues (Figure 4n–r). The effect of exosomal miR‐21‐3p on the M1 polarisation of AMs was further confirmed by fluorescent double staining of CD86 (marking AMs) and iNOS (marking M1‐type AMs) (Figure 4s). These data indicated that the expression of miR‐21‐3p in the lung tissue of SAP rats changed with the injection of exosomes from different treatment groups, which was consistent with the degree of lung injury. In conclusion, serum‐derived exosomal miR‐21‐3p is one of the causes of ALI during SAP, and EMO can alleviate ALI by reducing exosomal miR‐21‐3p and inhibiting the M1 polarisation of AMs.

### 3.5 Serum‐Derived Exosomes Promote M1 Polarisation of AMs via miR‐21‐3p In Vitro

**FIGURE 5:**
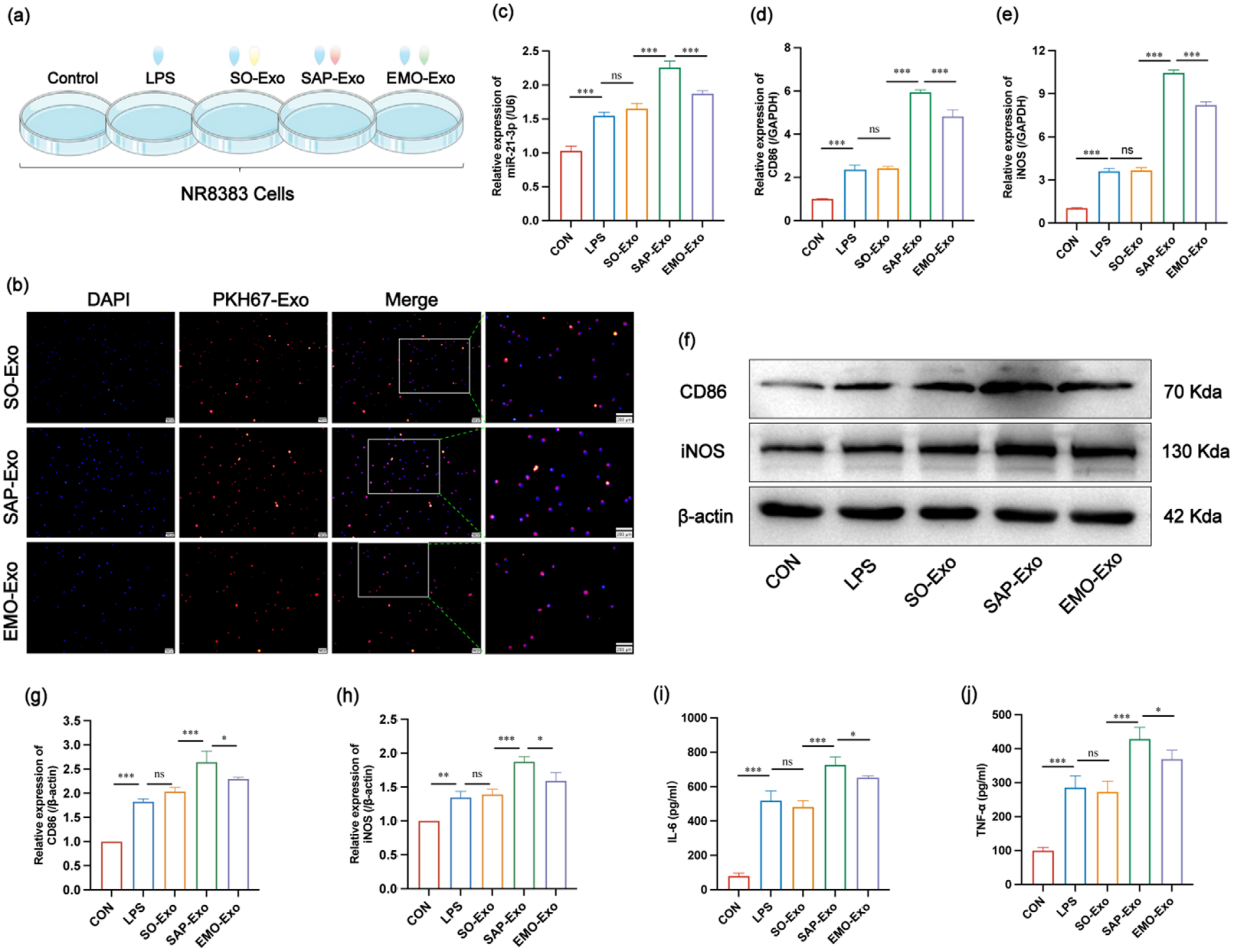
Serum‐derived exosomes promote M1 polarisation of AMs via miR‐21‐3p in vitro. (a) Schematic representation of NR8383 grouping and treatment. (b) Tracing of PKH‐26 fluorescently labelled exosomes in NR8383 cells (scale bar: 100 μm). (c) Gene expression levels of miR‐21‐3p in each group of cells. (d, e) Gene expression levels of CD86 and iNOS in each group of cells. (f) WB results of CD86 and iNOS in NR8383 cells of each group. (g, h) Relative quantification of CD86 and iNOS protein expression in each group of cells (*n* = 3). (i, j) Expression levels of IL‐6 and TNF‐α in cell culture supernatants in each group of cells.

In animals, we demonstrated that EMO attenuates SAP‐ALI by inhibiting M1 polarisation of AMs via a pathway that reduces serum‐derived exosomal miR‐21‐3p. To confirm this, we co‐cultured exosomes from each treatment group with NR8383 cells. The experimental groups and treatments were performed as shown in Figure 5a.

First, to confirm that exosomes were internalised by NR8383 cells, we co‐cultured PKH67 fluorescently labelled exosomes. As shown in Figure 5b, red fluorescently labelled exosomes were successfully internalised after co‐culturing with NR8383 for 24 h. Next, we measured the cellular miR‐21‐3p expression to verify the effect of exosomes on miR‐21‐3p expression in each treatment group. The expression of miR‐21‐3p in LPS‐stimulated NR8338 cells was higher than that in the CON group, and there was no significant difference between the SO‐Exo group and LPS stimulation alone. However, the expression of miR‐21‐3p in NR8338 cells in the SAP‐Exo group was elevated in the SO‐Exo group and decreased in the EMO‐Exo group compared to that in the SAP‐Exo group (Figure 5c). In addition, to confirm the degree of M1 macrophage polarisation, the mRNA and protein expression levels of CD86 and iNOS and the secretion of inflammatory cytokines (IL‐6 and TNF‐α) in the cell supernatant were detected. The results showed that the mRNA and protein expression levels of CD86 and iNOS in the LPS group increased, and there was no significant difference between the SO‐Exo and LPS groups. The SAP‐Exo group had significantly higher expression levels than in the SO‐Exo group, whereas these levels were lower in the EMO‐Exo group than in the SAP‐Exo group (Figure 5d,e). IL‐6 and TNF‐α in the supernatant showed the same trend (Figure 5f,g).

### 3.6 miR‐21‐3p Targets PTEN in NR8383 Cells

Previous studies have shown that PTEN plays an important role in the occurrence and development of ALI. The miRNA target gene database miRwalk (http://mirwalk.umm.uni‐heidelberg.de/) was used to predict the binding of miR‐21‐3p to PTEN. In addition, studies have confirmed that in both humans and mice, miR‐21‐3p can target and bind to PTEN [39, 40]. Therefore, we hypothesised that miR‐21‐3p promotes the development of SAP‐ALI by targeting PTEN.

**FIGURE 6:**
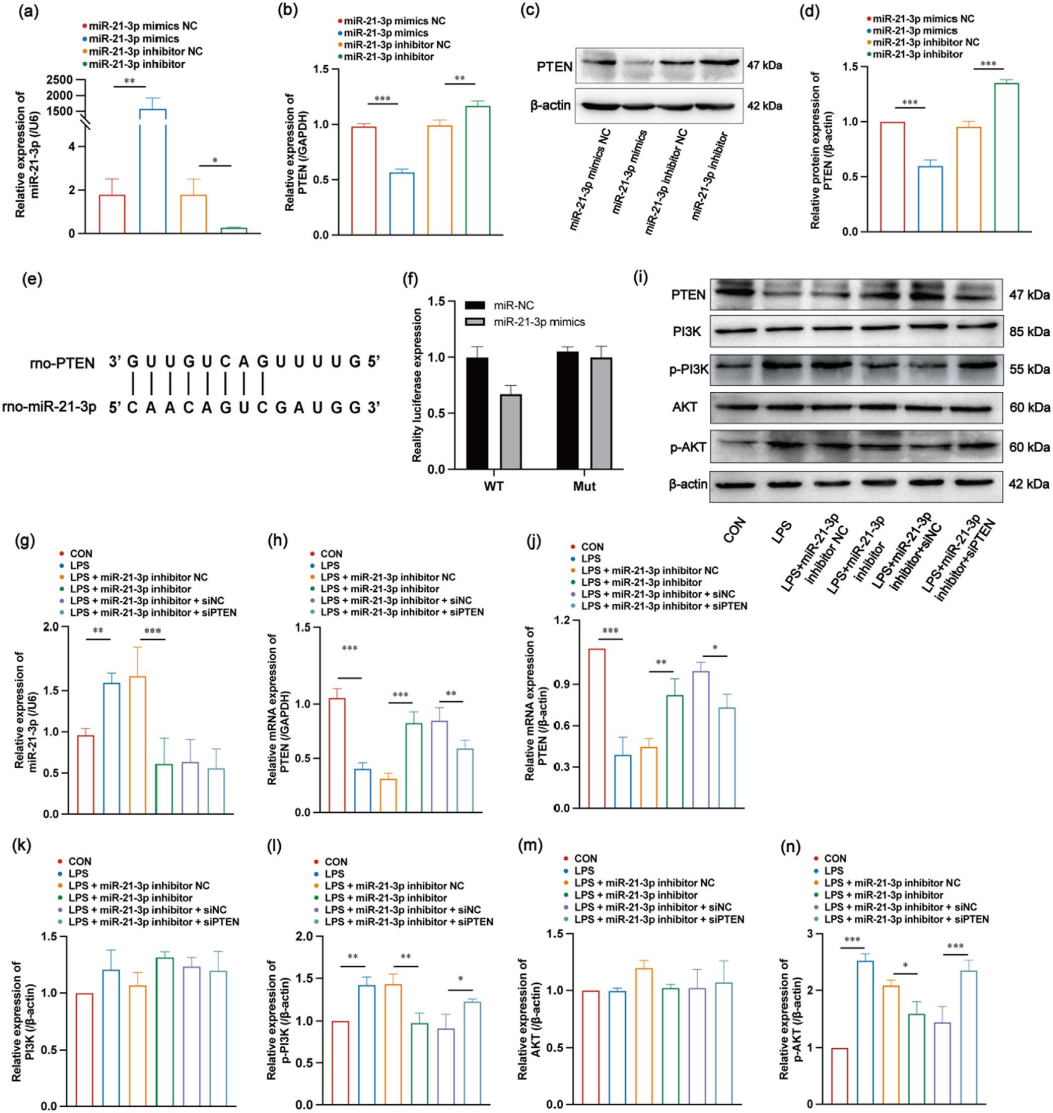
Targeting relationship between miR‐21‐3p and PTEN and mechanism by which miR‐21‐3p promotes M1 polarisation of AMs through PTEN/PI3K/AKT signalling pathway in vitro. (a, b) Gene expression levels of miR‐21‐3p and PTEN after transfection with miR‐21‐3p mimics and inhibitor. (c, d) WB and relative quantification results of PTEN after transfection with miR‐21‐3p mimics and inhibitor. **(e)** Prediction of binding sites between PTEN gene promoter region and miR‐361‐5p. **(f)** Binding of miR‐21‐3p to PTEN verified by dual luciferase reporter gene assay in NR8383 cells. (g) Gene expression levels of miR‐21‐3p in each group of cells. (h) Relative mRNA levels of PTEN. (i–l) Relative protein expression of PTEN, PI3K, p‐PI3K, AKT and p‐AKT in each group of cells. PTEN, PI3K, p‐PI3K, AKT and p‐AKT in each group (*n* = 3).

miR‐21‐3p overexpression and knockdown were achieved by transfecting miR‐21‐3p mimics and an miR‐21‐3p inhibitor, respectively, into NR8383 cells. The mRNA and protein expression levels of the downstream target gene, PTEN, were detected using RT‐qPCR and WB, respectively, to validate the effect of miR‐21‐3p on the PTEN‐targeting relationship. As shown in Figure 6a, transfection of miR‐21‐3p mimics and miR‐21‐3p inhibitor successfully overexpressed and knocked down miR‐21‐3p in NR8383 cells, compared to transfection with NC. Overexpression of miR‐21‐3p led to lower levels of PTEN mRNA and protein compared to the NC group, whereas inhibition of miR‐21‐3p increased the levels of PTEN mRNA and protein (Figure 6b–d). To further verify the direct binding of miR‐21‐3p to PTEN, we used RNAhybrid 2.2 to predict their binding sites (Figure 6e), constructed plasmids with WT or MUT 3′ UTR of PTEN, and co‐transfected them with miR‐21‐3p mimics or NC for dual luciferase reporter assay. We found that miR‐21‐3p mimics significantly reduced the luciferase reporter activity of the WT 3′ UTR plasmid of PTEN but not the MUT 3′ UTR plasmid (Figure 6f). These results indicate that the upregulation of miR‐21‐3p could downregulate the expression of PTEN.

### 3.7 miR‐21‐3p Targets PTEN to Activate PI3K/AKT Signalling Pathway Causing M1 Polarisation of AMs

We successfully verified that elevated miR‐21‐3p expression resulted in the downregulation of PTEN. However, whether the miR‐21‐3p‐induced M1 polarisation of AMs is regulated by PTEN remains unclear. Therefore, we performed dual intervention with miR‐21p and PTEN to further investigate the mechanism of miR‐21‐3p‐induced M1 polarisation in AMs. The results showed that compared to the CON group, the expression of miR‐21‐3p in the LPS group showed an upward trend, and the expression of PTEN showed a downward trend. After treatment with the miR‐21‐3p inhibitor based on LPS, the expression levels of miR‐21‐3p decreased and that of PTEN increased. The administration of siPTEN in combination with the miR‐21‐3p inhibitor reversed the effect of the miR‐21‐3p inhibitor alone on PTEN (Figure 6g,h,j). In addition, the expression of the PI3K/AKT signalling pathway also changed with PTEN, and WB results confirmed that PTEN had a negative regulatory effect on PI3K/AKT (Figure 6i,k–n).

**FIGURE 7:**
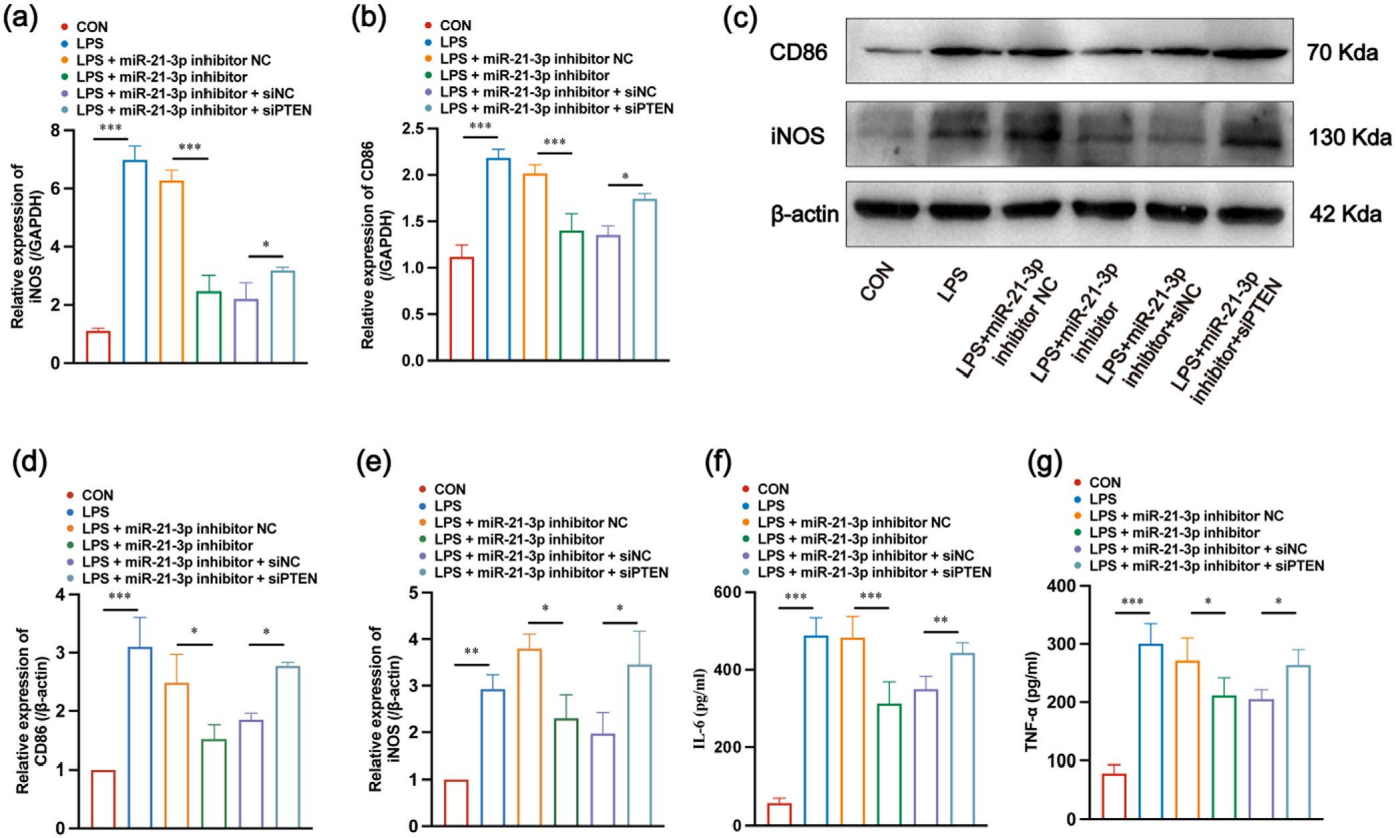
Mechanical process of miR‐21‐3p promotes M1 polarisation of AMs in vitro. (a, b) Gene expression levels of iNOS and CD86 in each group of cells using RT‐qPCR. (c–e) WB and relative quantification results of CD86 and iNOS in each experimental group (*n* = 3). (f, g) expression levels of IL‐6 and TNF‐α in cell culture supernatants of each group.

Next, we verified relevant indicators of M1 AM polarisation. RT‐qPCR and WB showed that the mRNA and protein levels of CD86 and iNOS were higher in the LPS group than in the CON group. After treatment with an miR‐21‐3p inhibitor based on LPS, the mRNA and protein levels of CD86 and iNOS were subsequently decreased. To verify whether the promotional effect of miR‐21‐3p on M1 polarisation of AMs was mediated by PTEN, we inhibited miR‐21‐3p while administering siPTEN to suppress PTEN expression. Administration of siPTEN, along with inhibition of miR‐21‐3p expression, reversed the inhibition of miR‐21‐3p alone, with relatively elevated mRNA levels of CD86 and iNOS (Figure 7a–e). The inflammatory factors IL‐6 and TNF‐α in the supernatant of cell culture also showed the same trend (Figure 7f,g). Taken together, these results indicate that miR‐21‐3p regulates the M1 polarisation of AMs by targeting PTEN/PI3K/AKT signalling.

## 4 Discussion

Our findings show that EMO may be therapeutic in SAP‐ALI by regulating serum‐derived exosomal miR‐21‐3p. To explore the mechanism of action of exosomes loaded with miR‐21‐3p, we injected exosomes with different levels of miR‐21‐3p in each group into rats via the tail vein or cocultured them with the alveolar macrophage cell line NR8383. We found that serum‐derived exosomes in the SAP group increased the expression levels of miR‐21‐3p in rat lung tissues and NR8383 cells and promoted M1 polarisation of AMs. In contrast, the effect of exosomes in the EMO group was attenuated compared to that in the SAP group. To further understand the role of miR‐21‐3p in macrophage polarisation, we knocked down the gene by chemical transfection of NR8383 cells. We found that elevated miR‐21‐3p expression suppressed the expression of the downstream target gene PTEN, activated the PI3K/AKT signalling pathway, and promoted M1 polarisation of AMs.

SAP is a common acute abdominal inflammatory disease in clinical practice, with morbidity and mortality rates as high as 35% [41]. MODS caused by SAP is the main cause of the high morbidity and mortality rates. The lungs are one of the most common sites of complications associated with SAP. EMO is an anthraquinone derivative of the TCM rhubarb, which has been shown to attenuate SAP‐ALI by interfering with the systemic inflammatory response and intestinal immune barrier [22, 29, 42, 43]. Macrophages residing in the tissues are important effector cells in these pathways. There are three types of macrophages in the lung tissues: airway, alveolar and pulmonary interstitial macrophages, with alveolar macrophages predominating (approximately 55%). When the lungs are stimulated by injury, circulating monocytes migrate into the lungs, develop into macrophages and progress to M1 macrophages, which release pro‐inflammatory mediators that induce lung inflammatory responses [44]. Related studies have shown that ALI induced by LPS, bleomycin and sepsis is accompanied by increased M1 polarisation of alveolar macrophages [45, 46]. Jiao et al. revealed that EMO inhibits the growth of hepatocellular carcinoma by inhibiting the conversion of hepatic M2 macrophages to M1 macrophages [47]. Furthermore, the combination of pseudoephedrine and EMO ameliorated LPS‐induced ALI by inhibiting the M1 polarisation of AMs [48]. Therefore, it should be determined whether EMO can treat SAP‐ALI by modulating M1 polarisation of AMs and its regulatory mechanisms.

Exosomes are extracellular vesicles from which all living cells secrete various bioactive components. Under inflammatory conditions, the number of exosomes secreted by cells increases and the bioactive components they carry are eventually transferred to distant target cells through the circulatory system to achieve distant secretory regulation [31, 49]. Alesanco et al. found that two exosome populations are produced during SAP: one exosome population of pancreatic origin enters the liver through the portal vein, and the liver produces another exosome population for release into the bloodstream [50]. Circulating exosomes can reach the alveolar compartments via blood transport and promote AMs towards a proinflammatory phenotype. A recent study showed that the treatment of SAP‐ALI by EMO partially depended on the exosomal pathway. Specifically, EMO was shown to reduce the production of harmful pancreatic‐derived exosomes and alter plasma exosomal protein composition, thereby modulating the PPARγ/NF‐κB pathway to reduce macrophage M1 polarisation in the lung [29]. However, detailed and specific mechanisms have not yet been elucidated. Recently, several herbal compounds and monomers have been shown to inhibit cellular inflammatory phenotypes via the exosomal miRNA pathway, thereby alleviating disease severity [51, 52]. Therefore, in this study, we focused on the exosomal miRNA pathway to elucidate the potential mechanisms by which emodin attenuates SAP‐ALI.

miRNAs are endogenous small molecule RNAs, which can bind to the 3′ UTR of target genes to cause gene silencing of target genes at the posttranscriptional level. Previous studies have shown that miRNAs are involved in regulating the progression of various acute lung inflammatory diseases including ARDS, SCAP and ASTHMA, etc., [53]. Among them, a variety of miRNAs such as miR‐1246, miR‐45, miR‐146a, miR‐193a‐3p and miR‐181a‐5p have been shown to play roles in regulating the polarisation of M1 macrophages. miR‐21 is the only miRNA overexpressed in any tumour, and its maturation includes the guide strand (miR‐21‐5p) and passenger strand (miR‐21‐3p) [54]. According to current reports, miR‐21‐3p is also an important miRNA involved in immune regulation in inflammatory diseases; it regulates intestinal immunity by targeting downstream MTDH and SORBS2 to modulate sepsis‐induced cardiac inflammation [37, 55]. In studies on AP and ALI, miR‐21‐3p was shown to be overexpressed in the acinar cells of AP mice [15]. When released into the blood, the plasma expression of miR‐21‐3p is positively correlated with the severity of AP and ALI in patients [17, 18]. These results suggested that miR‐21‐3p is an important gene in SAP‐ALI.

In this study, we first demonstrated that EMO was able to inhibit M1 polarisation of AMs to attenuate SAP‐ALI and that miR‐21‐3p expression in the lungs was positively correlated with the level of M1 polarisation of AMs, suggesting that EMO may attenuate SAP‐ALI by inhibiting the expression of miR‐21‐3p in lung tissues, thus inhibiting M1 polarisation of AMs. Notably, we detected miR‐21‐3p with the same expression trend in the lung tissue of serum‐derived exosomes from each treatment group. Based on the cellular interactions of exosomes, we hypothesised that EMO exerts its therapeutic effects by reducing exosomal miR‐21‐3p. To verify this hypothesis, we infused exosomes extracted from the serum of each treatment group back into rats with SAP via the tail vein to explore whether exosomes mediated the development of SAP‐ALI through the miR‐21‐3p pathway. The experimental results revealed that exosomes labelled with fluorescent material were concentrated in the lung tissues through the blood circulation. Injection of SO‐Exo did not significantly affect the severity of lung damage. However, the injection of SAP‐Exo considerably increased the levels of M1‐AMs and lung inflammation. In contrast, the injection of EMO‐Exo reduced the levels of M1‐AMs and lung inflammation compared to the injection of SAP‐Exo. The level of miR‐21‐3p was consistent with the M1 polarisation of AMs. To validate the effect of exosomal miR‐21‐3p on the M1 polarisation of AMs, we co‐cultured serum‐derived exosomes from each group with NR8338 cells. Fluorescence tracing showed that most of the cells absorbed serum‐derived exosomes after 24 h of co‐culture, and the expression level of miR‐21‐3p and the M1 polarisation levels of AMs in each group were consistent with the animal experimental results. Taken together, we tentatively confirmed that EMO could reduce the expression of serum‐derived exosomal miR‐21‐3p and cause AMs to express less miR‐21‐3p, thereby inhibiting M1 polarisation of AMs and reducing the severity of ALI.

Next, to confirm the mechanism by which miR‐21‐3p mediates M1 polarisation of AMs, we identified PTEN as the downstream target gene of miR‐21‐3p using miRNA target gene prediction combined with previous reports [39, 40, 56]. PTEN, an oncoprotein with dual‐specificity phosphatase activity, is a negative regulator of PI3K that dephosphorylates PIP3, thereby inhibiting AKT‐mediated signalling. PTEN inhibition blocks the activation of the PI3K/AKT signalling pathway, thereby reducing inflammation. Using miR‐21‐3p and PTEN loss‐of‐function experiments, we confirmed that elevated miR‐21‐3p expression inhibited downstream PTEN expression, which activated the PI3K/AKT signalling pathway, causing macrophage M1 polarisation. In the presence of elevated miR‐21‐3p levels, inhibition of downstream PTEN attenuated the expression of the PI3K/AKT signalling pathway, thereby reversing the increase in M1 polarisation of AMs caused by elevated miR‐21‐3p levels. Thus, PTEN, a target gene, is critical for the M1 polarisation of AMs, and miR‐21‐3p targets the PTEN/PI3K/AKT signalling pathway to induce M1 polarisation of AMs.

However, the present study has some limitations. First, the experimental sample size was small and clinical samples were not tested. Secondly, exosomal miRNAs constantly change during disease progression. We identified the expression of miR‐21‐3p in serum‐derived exosomes in a 24‐h SAP model but did not detect its dynamic changes during disease progression. It is also worth discussing when miR‐21‐3p levels are elevated and when they reach a peak. Third, thousands of miRNAs within exosomes can change during disease onset, and we have yet to explore other miRNAs. Fourth, miRNAs usually target multiple downstream genes, and it is worth exploring whether other target genes of miR‐21‐3p are involved in disease progression. Finally, the NR8383 cells were derived from rat AMs. Although they have promising applications in the study of lung diseases, their differences from the human body may limit the accurate understanding of human disease mechanisms.

## 5 Conclusions

We demonstrated that EMO has a therapeutic effect on SAP‐ALI. This effect is, in part, mediated by the inhibition of the expression of serum‐derived exosomal miR‐21‐3p, which modulates the PTEN/PI3K/AKT signalling pathway, ultimately leading to a decrease in the number of M1 polarisation of AMs and a reduction in the secretion of associated inflammatory cytokines. In addition, the exosomal miRNA pathway is one of the pathways through which EMO acts. In conclusion, EMO protects against SAP‐ALI and is a promising therapeutic strategy. This study provides a new theoretical basis for the treatment of SAP‐ALI.

## Author Contributions

**Bowen Lan:** methodology (lead), validation (lead), writing – original draft (lead). **Xuanchi Dong:** methodology (equal), writing – original draft (equal). **Qi Yang:** writing – review and editing (equal). **Haiyun Wen:** data curation (equal), validation (equal). **Yibo Zhang:** validation (equal). **Fan Li:** validation (equal). **Yinan Cao:** validation (equal). **Zhe Chen:** validation (equal). **Hailong Chen:** conceptualization (lead), funding acquisition (lead), project administration (lead), supervision (lead), writing – review and editing (lead).

## Conflicts of Interest

The authors declare no conflicts of interest.

## Data Availability

The data that support the findings of this study are available from the corresponding author upon reasonable request.
